# Supplementary material for: Unraveling new characteristics of γδ T cells using scRNA-seq in TCR KO chicken
Source: BMC Genomics. 2026 Mar 11;27:361. doi: 10.1186/s12864-026-12741-8 (PMC13063687; doi:10.1186/s12864-026-12741-8)
Supplement: Supplementary file 1 — Supplementary Material 1. [file 12864_2026_12741_MOESM1_ESM.pdf]

Supplementary Material

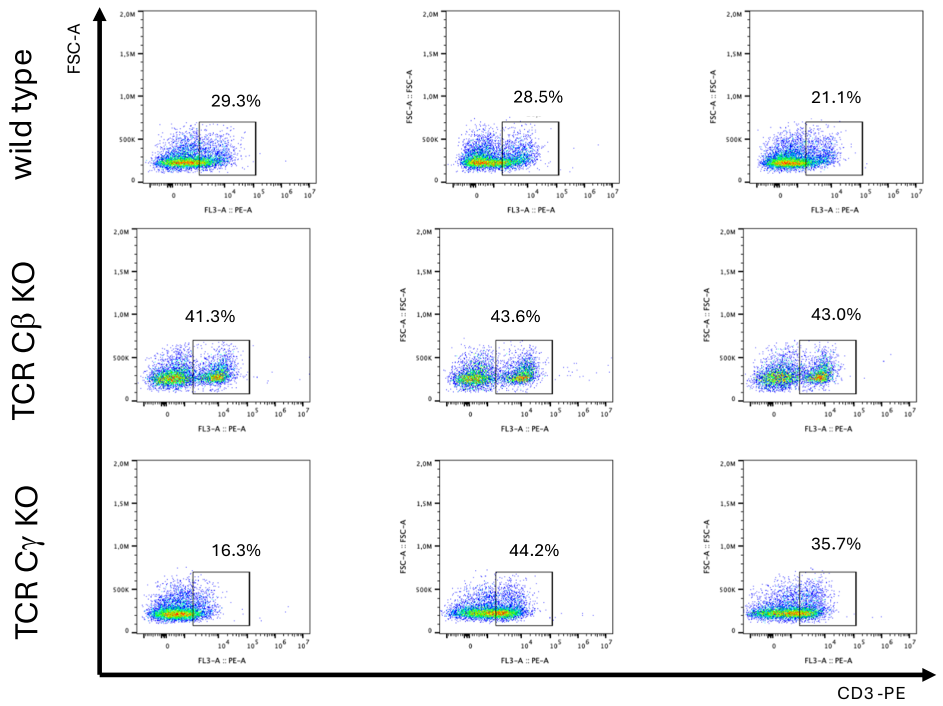

Supplementary Figure 1. Schematic representation of the flow-cytometry gating strategy for $CD3^+$ cell percentages in thymus. Flow-cytometry analysis of $CD3^+$ (CD3-PE) within single cells in percentages from wild type, TCR Cβ KO and TCR C γ KO at ED18.

A

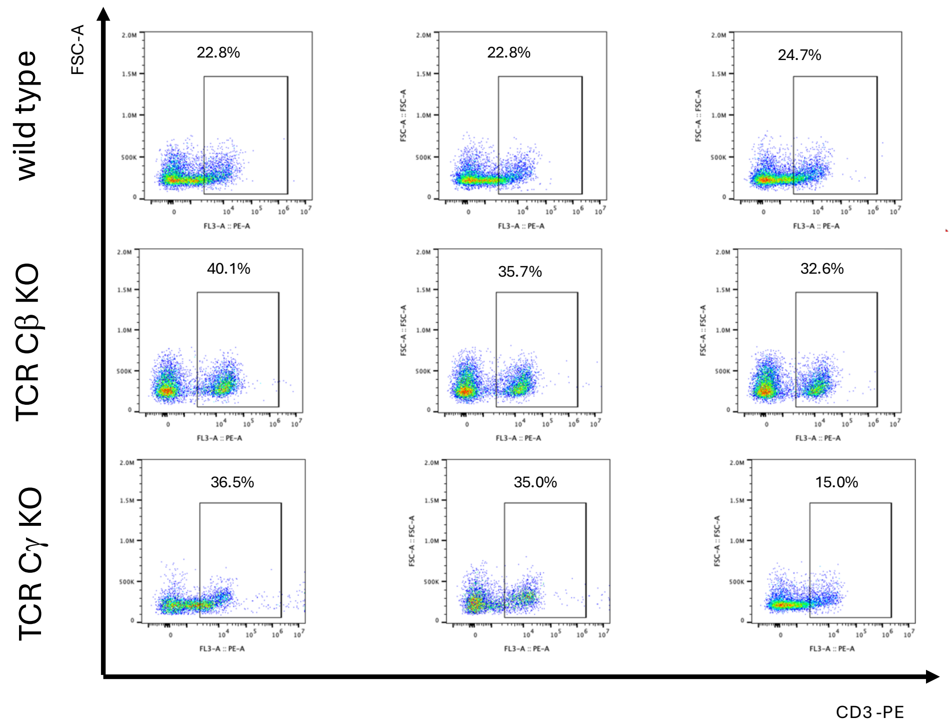

B

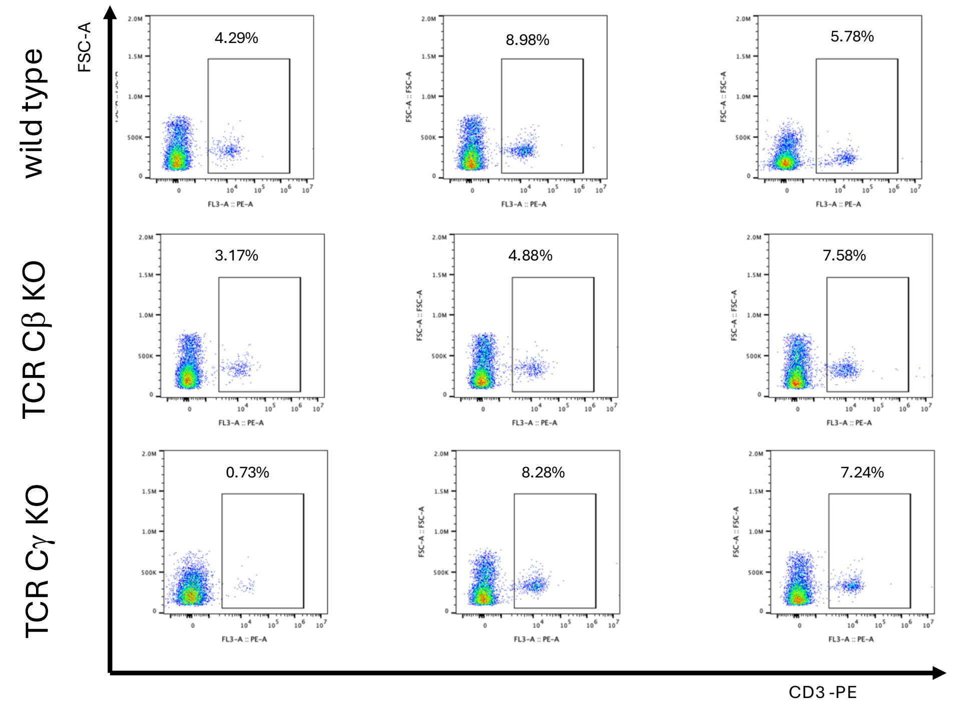

Supplementary Figure 2. Schematic representation of the flow-cytometry gating strategy for $CD3^+$ cell percentages in A) thymus and B) PBMCs. Flow-cytometry analysis of $CD3^+$ (CD3-PE) within single cells in percentages from wild type, TCR Cβ KO and TCR C γ KO at d3.

A

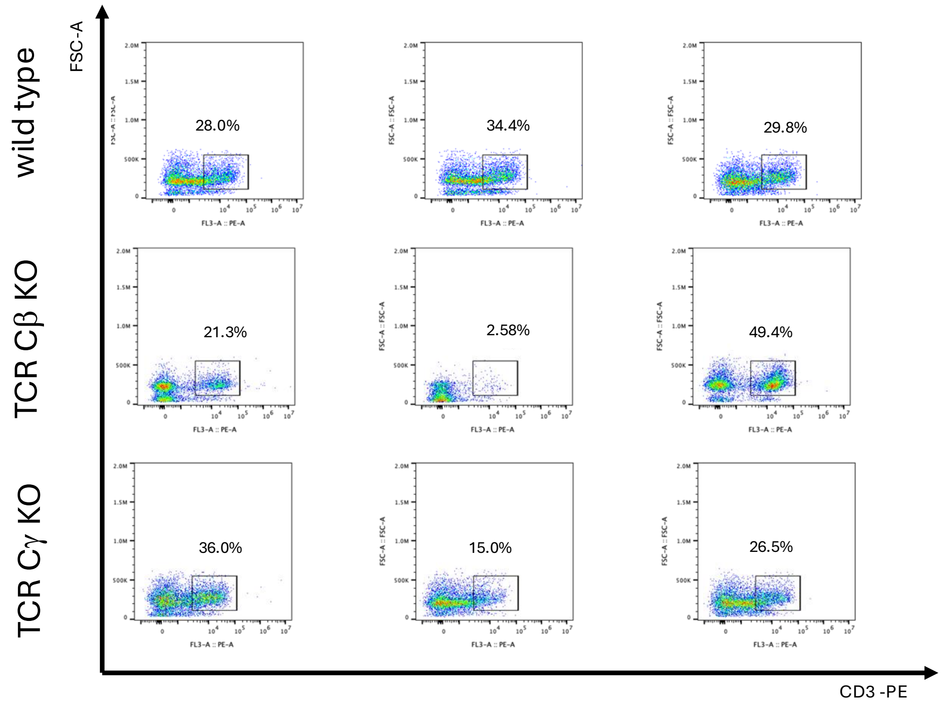

B

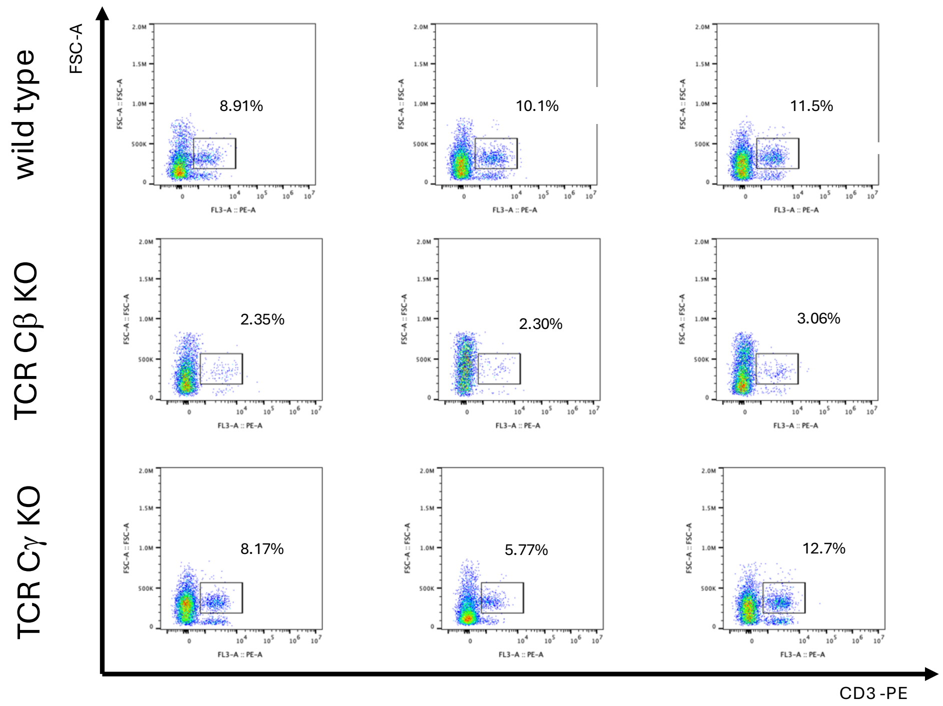

Supplementary Figure 3. Schematic representation of the flow-cytometry gating strategy for $CD3^+$ cell percentages in A) thymus and B) PBMCs. Flow-cytometry analysis of $CD3^+$ (CD3-PE) within single cells in percentages from wild type, TCR Cβ KO and TCR C γ KO at d14.

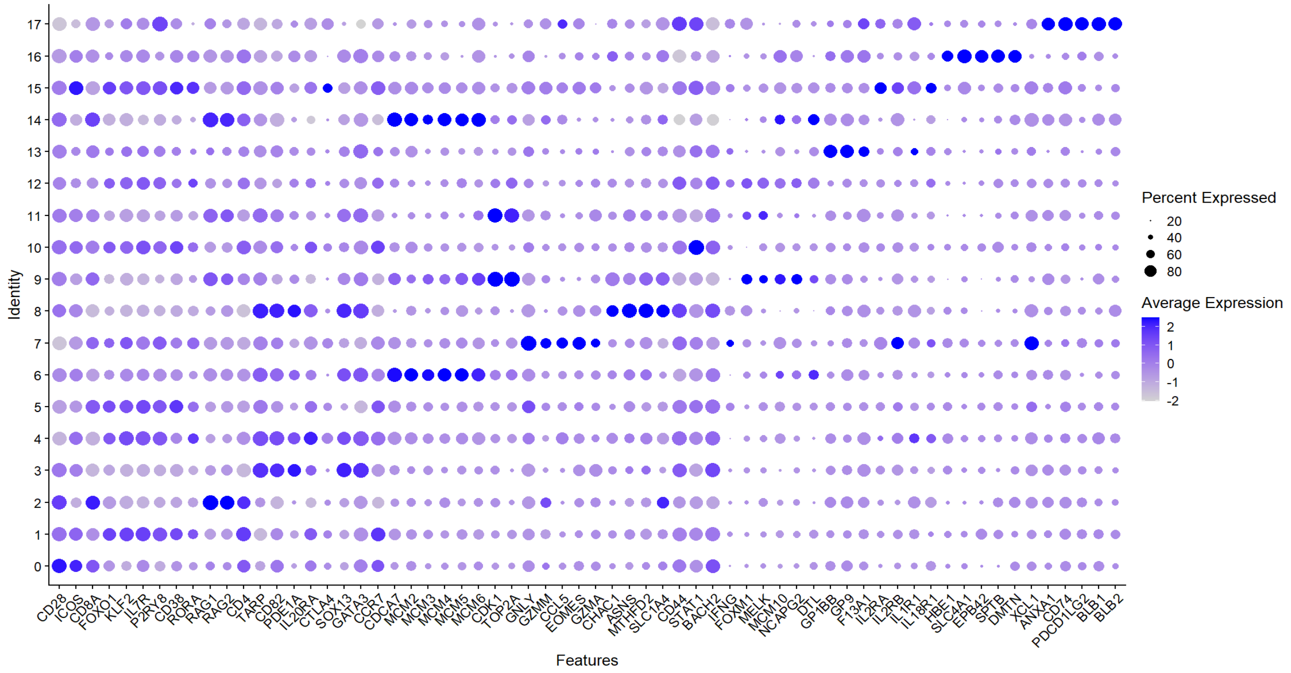

Supplementary Figure 4. Dot plot representing the expression of a selection of T cell associated genes in the integrated Clusters 0-17. The radius of the dot corresponds to the percentage of cells in each cluster expressing the gene, and color intensity corresponds to scaled expression values (average_log2 fold change).

Supplementary Table 1. Cellcount and percentages of each cluster from TCR Cβ KO, TCR Cγ Ko and WT animals on ED18, d3 and d14 in thymus

Cluster	Cellcount	Group
0	131	d14_TCRCbKO_PBMCs
1	921	d14_TCRCbKO_PBMCs
2	140	d14_TCRCbKO_PBMCs
3	35	d14_TCRCbKO_PBMCs
4	1175	d14_TCRCbKO_PBMCs
5	819	d14_TCRCbKO_PBMCs
6	135	d14_TCRCbKO_PBMCs
7	1345	d14_TCRCbKO_PBMCs
8	7	d14_TCRCbKO_PBMCs
9	37	d14_TCRCbKO_PBMCs
10	520	d14_TCRCbKO_PBMCs
11	64	d14_TCRCbKO_PBMCs
12	177	d14_TCRCbKO_PBMCs
13	178	d14_TCRCbKO_PBMCs
14	10	d14_TCRCbKO_PBMCs
15	68	d14_TCRCbKO_PBMCs
16	25	d14_TCRCbKO_PBMCs
17	19	d14_TCRCbKO_PBMCs
0	137	d14_TCRCgKO_PBMCs
1	2542	d14_TCRCgKO_PBMCs
2	154	d14_TCRCgKO_PBMCs
3	12	d14_TCRCgKO_PBMCs
4	90	d14_TCRCgKO_PBMCs
5	305	d14_TCRCgKO_PBMCs
6	98	d14_TCRCgKO_PBMCs
7	376	d14_TCRCgKO_PBMCs
8	9	d14_TCRCgKO_PBMCs
9	45	d14_TCRCgKO_PBMCs
10	364	d14_TCRCgKO_PBMCs
11	40	d14_TCRCgKO_PBMCs
12	173	d14_TCRCgKO_PBMCs
13	151	d14_TCRCgKO_PBMCs
14	9	d14_TCRCgKO_PBMCs
15	100	d14_TCRCgKO_PBMCs
16	14	d14_TCRCgKO_PBMCs
17	14	d14_TCRCgKO_PBMCs
0	160	d14_WT_PBMCs
1	1611	d14_WT_PBMCs
2	155	d14_WT_PBMCs

3	38	d14_WT_PBMCs
4	1018	d14_WT_PBMCs
5	381	d14_WT_PBMCs
6	138	d14_WT_PBMCs
7	661	d14_WT_PBMCs
8	5	d14_WT_PBMCs
9	83	d14_WT_PBMCs
10	208	d14_WT_PBMCs
11	50	d14_WT_PBMCs
12	294	d14_WT_PBMCs
13	184	d14_WT_PBMCs
14	11	d14_WT_PBMCs
15	87	d14_WT_PBMCs
16	25	d14_WT_PBMCs
17	11	d14_WT_PBMCs
0	76	d3_TCRCbKO_PBMCs
1	552	d3_TCRCbKO_PBMCs
2	57	d3_TCRCbKO_PBMCs
3	14	d3_TCRCbKO_PBMCs
4	482	d3_TCRCbKO_PBMCs
5	415	d3_TCRCbKO_PBMCs
6	47	d3_TCRCbKO_PBMCs
7	142	d3_TCRCbKO_PBMCs
8	2	d3_TCRCbKO_PBMCs
9	23	d3_TCRCbKO_PBMCs
10	86	d3_TCRCbKO_PBMCs
11	38	d3_TCRCbKO_PBMCs
12	31	d3_TCRCbKO_PBMCs
13	73	d3_TCRCbKO_PBMCs
14	1	d3_TCRCbKO_PBMCs
15	24	d3_TCRCbKO_PBMCs
16	30	d3_TCRCbKO_PBMCs
17	13	d3_TCRCbKO_PBMCs
0	145	d3_TCRCgKO_PBMCs
1	2150	d3_TCRCgKO_PBMCs
2	52	d3_TCRCgKO_PBMCs
3	2	d3_TCRCgKO_PBMCs
4	125	d3_TCRCgKO_PBMCs
5	292	d3_TCRCgKO_PBMCs
6	163	d3_TCRCgKO_PBMCs
7	83	d3_TCRCgKO_PBMCs
8	1	d3_TCRCgKO_PBMCs
9	38	d3_TCRCgKO_PBMCs

10	113	d3_TCRCgKO_PBMCs
11	63	d3_TCRCgKO_PBMCs
12	126	d3_TCRCgKO_PBMCs
13	79	d3_TCRCgKO_PBMCs
14	1	d3_TCRCgKO_PBMCs
15	247	d3_TCRCgKO_PBMCs
16	31	d3_TCRCgKO_PBMCs
17	14	d3_TCRCgKO_PBMCs
0	145	d3_WT_PBMCs
1	1784	d3_WT_PBMCs
2	93	d3_WT_PBMCs
3	11	d3_WT_PBMCs
4	525	d3_WT_PBMCs
5	531	d3_WT_PBMCs
6	173	d3_WT_PBMCs
7	77	d3_WT_PBMCs
8	4	d3_WT_PBMCs
9	39	d3_WT_PBMCs
10	153	d3_WT_PBMCs
11	88	d3_WT_PBMCs
12	68	d3_WT_PBMCs
13	98	d3_WT_PBMCs
14	11	d3_WT_PBMCs
15	252	d3_WT_PBMCs
16	17	d3_WT_PBMCs
17	5	d3_WT_PBMCs
0	154	d3_TCRCbKO_Thymus
1	125	d3_TCRCbKO_Thymus
2	82	d3_TCRCbKO_Thymus
3	737	d3_TCRCbKO_Thymus
4	167	d3_TCRCbKO_Thymus
5	165	d3_TCRCbKO_Thymus
6	375	d3_TCRCbKO_Thymus
7	35	d3_TCRCbKO_Thymus
8	507	d3_TCRCbKO_Thymus
9	203	d3_TCRCbKO_Thymus
10	48	d3_TCRCbKO_Thymus
11	158	d3_TCRCbKO_Thymus
12	72	d3_TCRCbKO_Thymus
13	52	d3_TCRCbKO_Thymus
14	23	d3_TCRCbKO_Thymus
15	23	d3_TCRCbKO_Thymus
16	8	d3_TCRCbKO_Thymus

17	5	d3_TCRCbKO_Thymus
0	1764	d3_TCRCgKO_Thymus
1	859	d3_TCRCgKO_Thymus
2	429	d3_TCRCgKO_Thymus
3	10	d3_TCRCgKO_Thymus
4	31	d3_TCRCgKO_Thymus
5	211	d3_TCRCgKO_Thymus
6	134	d3_TCRCgKO_Thymus
7	36	d3_TCRCgKO_Thymus
8	10	d3_TCRCgKO_Thymus
9	118	d3_TCRCgKO_Thymus
10	101	d3_TCRCgKO_Thymus
11	62	d3_TCRCgKO_Thymus
12	133	d3_TCRCgKO_Thymus
13	151	d3_TCRCgKO_Thymus
14	55	d3_TCRCgKO_Thymus
15	174	d3_TCRCgKO_Thymus
16	35	d3_TCRCgKO_Thymus
17	22	d3_TCRCgKO_Thymus
0	1459	d3_WT_Thymus
1	341	d3_WT_Thymus
2	744	d3_WT_Thymus
3	352	d3_WT_Thymus
4	68	d3_WT_Thymus
5	198	d3_WT_Thymus
6	222	d3_WT_Thymus
7	14	d3_WT_Thymus
8	168	d3_WT_Thymus
9	249	d3_WT_Thymus
10	49	d3_WT_Thymus
11	158	d3_WT_Thymus
12	64	d3_WT_Thymus
13	97	d3_WT_Thymus
14	132	d3_WT_Thymus
15	37	d3_WT_Thymus
16	16	d3_WT_Thymus
17	4	d3_WT_Thymus
0	606	ED18_TCRCbKO_Thymus
1	382	ED18_TCRCbKO_Thymus
2	439	ED18_TCRCbKO_Thymus
3	3313	ED18_TCRCbKO_Thymus
4	639	ED18_TCRCbKO_Thymus
5	447	ED18_TCRCbKO_Thymus

6	992	ED18_TCRCbKO_Thymus
7	47	ED18_TCRCbKO_Thymus
8	1369	ED18_TCRCbKO_Thymus
9	320	ED18_TCRCbKO_Thymus
10	74	ED18_TCRCbKO_Thymus
11	535	ED18_TCRCbKO_Thymus
12	216	ED18_TCRCbKO_Thymus
13	3	ED18_TCRCbKO_Thymus
14	104	ED18_TCRCbKO_Thymus
15	32	ED18_TCRCbKO_Thymus
16	2	ED18_TCRCbKO_Thymus
17	1	ED18_TCRCbKO_Thymus
0	3483	ED18_TCRCgKO_Thymus
1	178	ED18_TCRCgKO_Thymus
2	4979	ED18_TCRCgKO_Thymus
3	93	ED18_TCRCgKO_Thymus
4	3	ED18_TCRCgKO_Thymus
5	92	ED18_TCRCgKO_Thymus
6	63	ED18_TCRCgKO_Thymus
7	1	ED18_TCRCgKO_Thymus
8	42	ED18_TCRCgKO_Thymus
9	938	ED18_TCRCgKO_Thymus
10	45	ED18_TCRCgKO_Thymus
11	367	ED18_TCRCgKO_Thymus
12	106	ED18_TCRCgKO_Thymus
13	9	ED18_TCRCgKO_Thymus
14	561	ED18_TCRCgKO_Thymus
16	17	ED18_TCRCgKO_Thymus
17	1	ED18_TCRCgKO_Thymus
0	1959	ED18_WT_Thymus
1	171	ED18_WT_Thymus
2	1626	ED18_WT_Thymus
3	1412	ED18_WT_Thymus
4	300	ED18_WT_Thymus
5	268	ED18_WT_Thymus
6	466	ED18_WT_Thymus
7	18	ED18_WT_Thymus
8	558	ED18_WT_Thymus
9	624	ED18_WT_Thymus
10	86	ED18_WT_Thymus
11	367	ED18_WT_Thymus
12	144	ED18_WT_Thymus
13	5	ED18_WT_Thymus

14	320	ED18_WT_Thymus
15	17	ED18_WT_Thymus
16	3	ED18_WT_Thymus
17	1	ED18_WT_Thymus
0	409	d14_TCRCbKO_Thymus
1	322	d14_TCRCbKO_Thymus
2	170	d14_TCRCbKO_Thymus
3	780	d14_TCRCbKO_Thymus
4	461	d14_TCRCbKO_Thymus
5	400	d14_TCRCbKO_Thymus
6	187	d14_TCRCbKO_Thymus
7	156	d14_TCRCbKO_Thymus
8	434	d14_TCRCbKO_Thymus
9	104	d14_TCRCbKO_Thymus
10	442	d14_TCRCbKO_Thymus
11	127	d14_TCRCbKO_Thymus
12	95	d14_TCRCbKO_Thymus
13	139	d14_TCRCbKO_Thymus
14	32	d14_TCRCbKO_Thymus
15	47	d14_TCRCbKO_Thymus
16	20	d14_TCRCbKO_Thymus
17	9	d14_TCRCbKO_Thymus
0	1544	d14_TCRCgKO_Thymus
1	1130	d14_TCRCgKO_Thymus
2	560	d14_TCRCgKO_Thymus
3	18	d14_TCRCgKO_Thymus
4	51	d14_TCRCgKO_Thymus
5	331	d14_TCRCgKO_Thymus
6	109	d14_TCRCgKO_Thymus
7	233	d14_TCRCgKO_Thymus
8	7	d14_TCRCgKO_Thymus
9	136	d14_TCRCgKO_Thymus
10	385	d14_TCRCgKO_Thymus
11	74	d14_TCRCgKO_Thymus
12	194	d14_TCRCgKO_Thymus
13	161	d14_TCRCgKO_Thymus
14	66	d14_TCRCgKO_Thymus
15	79	d14_TCRCgKO_Thymus
16	26	d14_TCRCgKO_Thymus
17	11	d14_TCRCgKO_Thymus
0	1676	d14_WT_Thymus
1	762	d14_WT_Thymus
2	773	d14_WT_Thymus

3	503	d14_WT_Thymus
4	228	d14_WT_Thymus
5	495	d14_WT_Thymus
6	237	d14_WT_Thymus
7	253	d14_WT_Thymus
8	206	d14_WT_Thymus
9	182	d14_WT_Thymus
10	209	d14_WT_Thymus
11	127	d14_WT_Thymus
12	256	d14_WT_Thymus
13	190	d14_WT_Thymus
14	65	d14_WT_Thymus
15	42	d14_WT_Thymus
16	21	d14_WT_Thymus
17	8	d14_WT_Thymus
